# Supplementary material for: Adverse Events in Targeted Therapy for Unresectable Hepatocellular Carcinoma Predict Clinical Outcomes
Source: Cancers (Basel). 2024 Sep 14;16(18):3150. doi: 10.3390/cancers16183150 (PMC11430790; doi:10.3390/cancers16183150)
Supplement: Supplementary file 1 [file cancers-16-03150-s001.zip › cancers-3143532-supplementary.pdf]

Supplementary Figure S1. Overall survival (OS) and progression free survival (PFS) curves between first- and later-line groups.

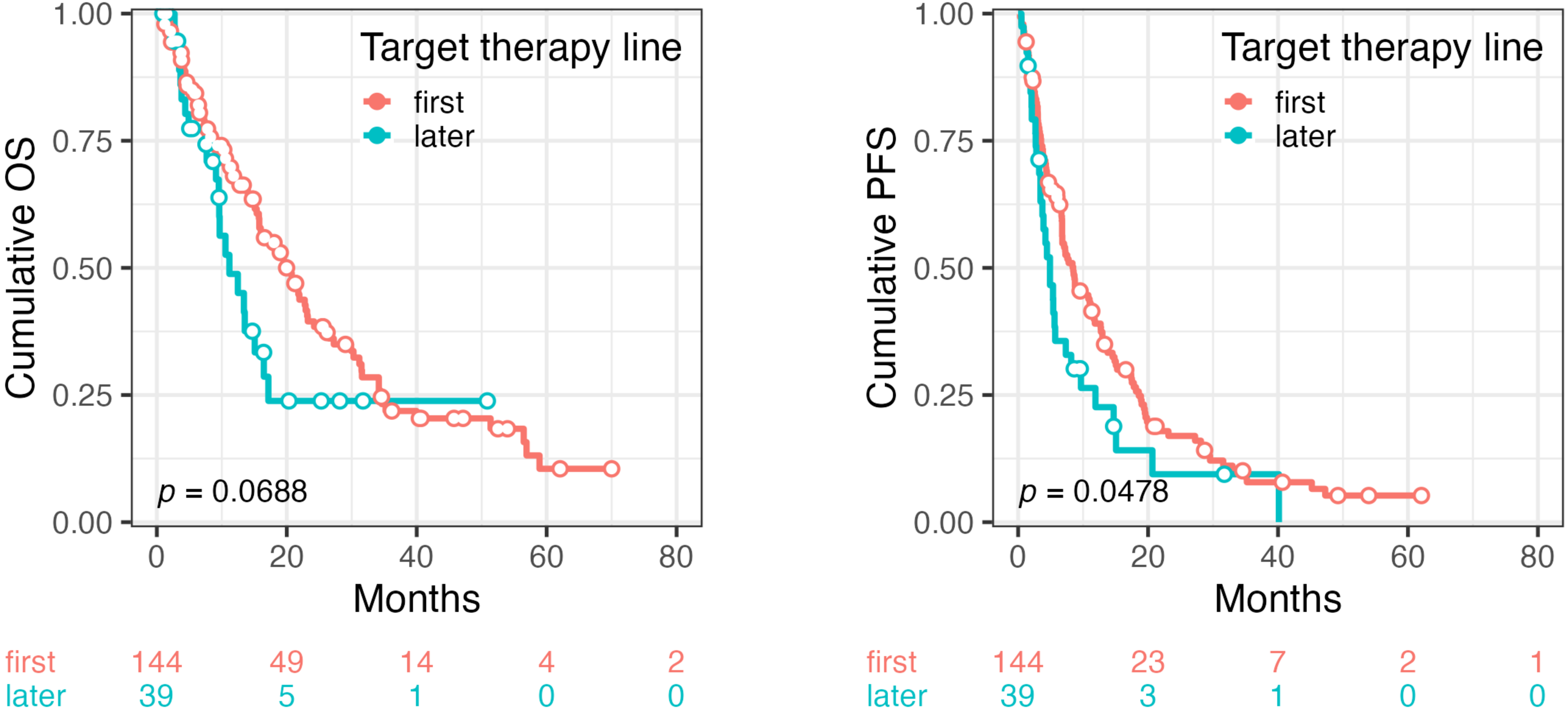

Supplementary Table S1. The number of anticancer drugs stratified by treatment line in this study.

	First-line	Later-line (Second/Third/Forth-line)
AB	30	10<br>(3/6/1)
LEN	40	17<br>(16/1/0)
SOR	73	6<br>(6/0/0)
CAB	0	3<br>(2/1/0)
RAM	1	2<br>(2/0/0)
REG	0	1<br>(1/0/0)

AB, atezolizumab plus bevacizumab ; LEN, lenvatinib; SOR, sorafenib; CAB, cabozantinib; RAM, ramucirumab; REG, regorafenib.

Supplementary Table S2. Adverse events between first- and later-line groups.

	First-line	Later-line	*p* value
Appetite loss (G0/1/2/3)	73/16/49/6	24/7/6/2	0.098
General fatigue (G0/1/2/3)	81/21/37/5	21/10/7/1	0.381
Hypertension (G0/1/2/3)	93/4/31/16	27/2/9/1	0.327
Hand-foot syndrome (G0/1/2/3)	103/16/23/2	32/5/1/1	0.095
Proteinuria (G0/1/2/3)	117/9/12/6	23/4/4/8	0.004
Hypothyroidism (G0/1/2)	122/7/15	26/5/8	0.027
